# Treatment of Hydrocele with Ioduretted Injections

**Published:** 1845-01

**Authors:** 


*Treatment of Hydrocele with Ioduretted Injections.*—In more than 300 cases of this complaint treated with an ioduretted injection, (composed of tincture of iodine 4 parts, and distilled water 125 parts,) by M. Velpeau, not a single accident or unpleasant symptom has ever occurred. One of the patients indeed died; but the fatal result in this instance proceeded from a purulent inflammation of the cellular tissue of the pelvis, quite unconnected with the operation, and not having any communication whatever with the affection of the scrotum. The average period for effecting the cure was 15 days. In one case only the injection found its way into the tissue of the scrotum, in place of the tunica vaginalis: notwithstanding this misadventure, no appearance of gangrene supervened, and the patient recovered without any unpleasant accident.—*Med. Chir. Rev., from L'Experience.*

